# Measuring therapeutic engagement in finnish adult acute psychiatric in-patient care units using the finnish version of therapeutic engagement questionnaire (TEQ)

**DOI:** 10.1192/j.eurpsy.2021.1042

**Published:** 2021-08-13

**Authors:** R. Askola, J. Turunen, A. Hottinen, X. Kantaris, M. Chambers, L. Kuosmanen

**Affiliations:** 1 Department Of Nursing Science, University of Eastern Finland, Kuopio, Finland; 2 Psychiatry, Helsinki University Hospital, Helsinki, Finland; 3 Faculty Of Health, Social Care And Education, Kingston University and St George’s, University of London, London, United Kingdom; 4 Faculty Of Health, Social Care And Education, Kingston and St. George’s, University of London, London, United Kingdom

**Keywords:** therapeutic engagement, mental health, acute psychiatry, interactions

## Abstract

**Introduction:**

The Therapeutic Engagement Questionnaire (TEQ) has been developed and validated in partnership with service users (SUs), registered mental health nurses (RMHNs) and nurse academics in the UK in accordance with psychometric theory. The TEQ is highly relevant and useful to clinical practice. The TEQ measures therapeutic engagement (TE) in two contexts - 1-1 interactions between SUs and RMHNs, as well as the overall environment and atmosphere of the units - from the perspective of both SUs and RMHNs. The TEQ has been translated into Finnish by two expert panels and was pre-tested and validated in ten adult acute psychiatric in-patient units in two hospitals in Finland.

**Objectives:**

To measure TE in Finnish adult acute in-patient psychiatric settings from the perspectives of both SUs and RMHNs.

**Methods:**

The Finnish version of the TEQ (Hoidollinen yhteistyö) will be completed by RMHNs and SUs in 15 adult acute psychiatric in-patient units. Nine of the units are within the University Hospital and six in a municipal psychiatric hospital. The data will be collected within a 3-month period (October - December 2020). The coordinating nurse of each unit will organise the operational side of the study including obtaining consent from SUs. The nurses will participate in the survey via Webropol which includes nurses’ consent. Sociodemographic information will be collected from the SUs and nurses.

**Results:**

The results of the measurement study will be reported at the 29th European Congress of Psychiatry.

**Conclusions:**

The conclusions of the measurement study will be reported at the 29th European Congress of Psychiatry.

**Conflict of interest:**

This study is supported by the National Institute for Health Research (NIHR) Applied Research Collaboration South London (NIHR ARC South London).

